# Endoscopic Rendezvous Technique as a Salvage Strategy for Incomplete Pancreas Divisum

**DOI:** 10.14309/crj.0000000000002396

**Published:** 2026-11-13

**Authors:** Rached Radwan, Dima Siblini, Rima Siblini, Abbas Rachid, Zaher Houmani

**Affiliations:** 1 Department of Gastroenterology and Hepatology, Lebanese University, Beirut, Lebanon; 2 Department of Internal Medicine, Lebanese University, Beirut, Lebanon; 3 Department of Internal Medicine, American University of Beirut, Beirut, Lebanon; 4 Department of Internal Medicine and Cardiology, Lebanese University, Beirut, Lebanon; 5 Department of Gastroenterology and Hepatology, AL Zahraa University Medical Center, Beirut, Lebanon

**Keywords:** incomplete pancreas divisum, recurrent pancreatitis, minor papilla, impacted stone, endoscopic retrograde cholangiopancreatography, endoscopic ultrasound, transpapillary rendezvous technique, case report

## Abstract

Pancreas divisum (PD) is the most common congenital anomaly of the pancreatic ductal system, resulting from incomplete fusion of the dorsal and ventral pancreatic ducts during embryogenesis and occurring in approximately 5%–10% of the general population. Although most individuals remain asymptomatic, a subset may develop recurrent acute pancreatitis, potentially related to impaired pancreatic drainage through the minor papilla. We report the case of a 55-year-old woman with a 12-year history of recurrent pancreatitis and persistent epigastric pain, in whom an incomplete PD with an impacted pancreatic duct stone was ultimately identified. Initial cross-sectional imaging failed to demonstrate the underlying ductal anomaly. Conventional minor papilla cannulation during endoscopic retrograde cholangiopancreatography was unsuccessful because of the small and edematous papillary orifice. A transpapillary rendezvous technique was subsequently employed to facilitate access to the dorsal pancreatic duct, allowing minor papilla sphincterotomy, stone extraction, and pancreatic duct stent placement. This case highlights the diagnostic challenges of incomplete PD and demonstrates a technically demanding endoscopic rescue strategy when conventional minor papilla cannulation fails.

## INTRODUCTION

The etiologies of recurrent pancreatitis are diverse and encompass a broad spectrum of underlying conditions, including biliary disease, sphincter of Oddi dysfunction, alcohol use, genetic predisposition, autoimmune pancreatitis, and structural abnormalities that impair pancreatic ductal drainage, including congenital pancreatic ductal variants and obstruction of the pancreatic or pancreatobiliary ducts.^1^

Identifying an underlying structural abnormality is particularly important in patients with recurrent or otherwise unexplained pancreatitis, as appropriate diagnosis and targeted intervention may reduce the risk of recurrence and progression to chronic pancreatic injury. Pancreas divisum (PD) is the most common congenital anomaly of the pancreatic ductal system and results from failure of fusion of the dorsal and ventral pancreatic ducts during embryologic development. Although the majority of individuals with PD remain asymptomatic, approximately 5% may develop recurrent episodes of acute pancreatitis, potentially related to impaired pancreatic juice drainage through the minor papilla.^2^

In susceptible individuals, relative obstruction at the minor papilla may contribute to recurrent pancreatic inflammation and, over time, progression toward chronic pancreatitis. Three anatomical variants of PD have been described: complete, incomplete, and reverse PD. Complete PD, the most common form, is characterized by complete failure of fusion between the dorsal and ventral ductal systems, with most pancreatic drainage occurring through the minor papilla. Incomplete PD is less common and is characterized by partial fusion with persistence of a small communicating branch between the dorsal and ventral ducts. Reverse PD is a rare variant in which the dorsal ductal system has limited or absent communication with the main pancreatic duct, resulting in an altered pattern of pancreatic drainage.^3^

Accurate recognition of pancreatic ductal anatomy is essential in patients with recurrent pancreatitis, particularly when conventional imaging fails to identify an underlying cause. Magnetic resonance cholangiopancreatography (MRCP), endoscopic ultrasound (EUS), and endoscopic retrograde cholangiopancreatography (ERCP) can provide complementary information regarding ductal anatomy and facilitate therapeutic intervention. However, incomplete PD may be particularly challenging to diagnose and manage because the persistent communication between the dorsal and ventral ductal systems can be subtle, while access to the minor papilla may be technically difficult.

We report a case of long-standing recurrent pancreatitis caused by previously unrecognized incomplete PD with an impacted pancreatic duct stone. The case highlights the diagnostic challenges associated with this uncommon ductal variant. It demonstrates a transpapillary rendezvous-assisted ERCP technique as a salvage strategy when conventional minor papilla cannulation is unsuccessful. A 55-year-old woman with a medical history of diabetes mellitus presented with a 12-year history of persistent epigastric pain radiating to the back, despite the use of multiple analgesic medications. Her symptoms were intermittently associated with vomiting and chills, without documented fever. She denied alcohol consumption and smoking. On presentation, her vital signs were stable, with a blood pressure of 110/60 mm Hg, heart rate of 77 beats/min, and no fever. There was no scleral icterus. Abdominal examination revealed a soft, nondistended abdomen with mild epigastric tenderness, without guarding, rebound tenderness, or other significant findings. Initial laboratory investigations were largely unremarkable, apart from a mild elevation in serum lipase. There was no evidence of leukocytosis, anemia, renal dysfunction, or cholestatic or hepatocellular injury. Detailed laboratory findings are presented in Table 1.

**Table 1.** Laboratory investigation of the patient during hospitalization

Parameter	Patient value	Reference range
WBC, per mm^3^	9,000	4,500–11,000
Hemoglobin, g/dL	13.9	13.5–17.5
Creatinine, mg/dL	0.6	<1

BUN	25 mg/dL	<20 mg/dL
Lipase	164 U/L	0–160 U/L
ALP	53 IU/L	44–147 IU/L

ALP, alkaline phosphatase; BUN, blood urea nitrogen; WBC, white blood cells.

Abdominal and pelvic contrast-enhanced computed tomography demonstrated multiple pancreatic parenchymal calcifications with extensive peripancreatic inflammatory changes and edema, consistent with chronic pancreatitis. Multiple calculi were identified within the proximal main pancreatic duct, the largest measuring 8 mm, with associated upstream pancreatic ductal dilatation of approximately 6 mm distal to the obstruction.

Subsequent magnetic resonance imaging (MRI) of the abdomen with MRCP confirmed multiple intraductal pancreatic calculi, including an 8 mm stone within the proximal pancreatic duct, with approximately 5 mm of upstream ductal dilatation. The study also identified a pancreatic tail pseudocyst, further supporting the diagnosis of chronic pancreatitis. Notably, the initial MRI/MRCP did not demonstrate any congenital pancreatic ductal anomaly.

The patient was not experiencing acute pancreatitis at the time of evaluation. However, given her longstanding and debilitating abdominal pain and recurrent episodes of pancreatitis, the most recent occurring 6 weeks earlier, EUS was performed to delineate the pancreatic ductal anatomy further and investigate a potential underlying structural abnormality. Careful tracing of the main pancreatic duct toward the papillary region suggested drainage through the minor papilla, without clear continuity with the ventral pancreatic duct. These findings raised suspicion for PD. Given the severity of her chronic abdominal pain, a celiac plexus block was performed at the conclusion of the EUS using bupivacaine and triamcinolone, without immediate complications.

Given the presence of an impacted pancreatic duct stone and suspected PD, ERCP was subsequently performed for definitive ductal evaluation and therapeutic intervention. The minor papilla was markedly diminutive and appeared edematous, possibly reflecting increased intraductal pressure. Conventional cannulation of the minor papilla was unsuccessful, and repeated attempts to advance a guidewire into the dorsal pancreatic duct through the minor papilla were unsuccessful.

To overcome the difficulty in accessing the minor papilla, a retrograde transpapillary rendezvous technique was performed. The major papilla was successfully cannulated, and fluoroscopic pancreatography demonstrated findings consistent with PD. A guidewire was advance retrogradely toward the minor papilla through a small communicating branch between the ventral and dorsal pancreatic ducts, confirming incomplete PD (Figure 1). The duodenoscope was then used to facilitate completion of the rendezvous procedure (Figure 2). The duodenal end of the guidewire was grasped with biopsy forceps and retrieved through the minor papilla, thereby establishing access to the dorsal pancreatic duct (Figure 3).

**Figure 1.**
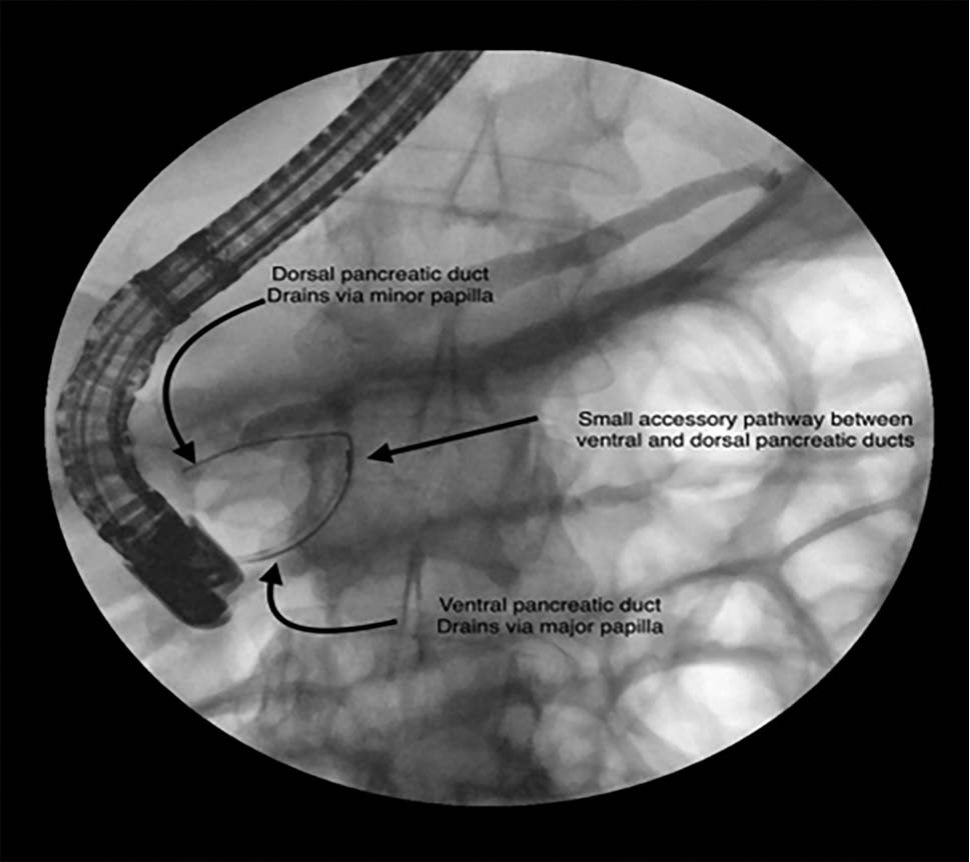
Fluoroscopic demonstration of incomplete pancreas divisum and retrograde guidewire passage through the communicating branch.

**Figure 2.**
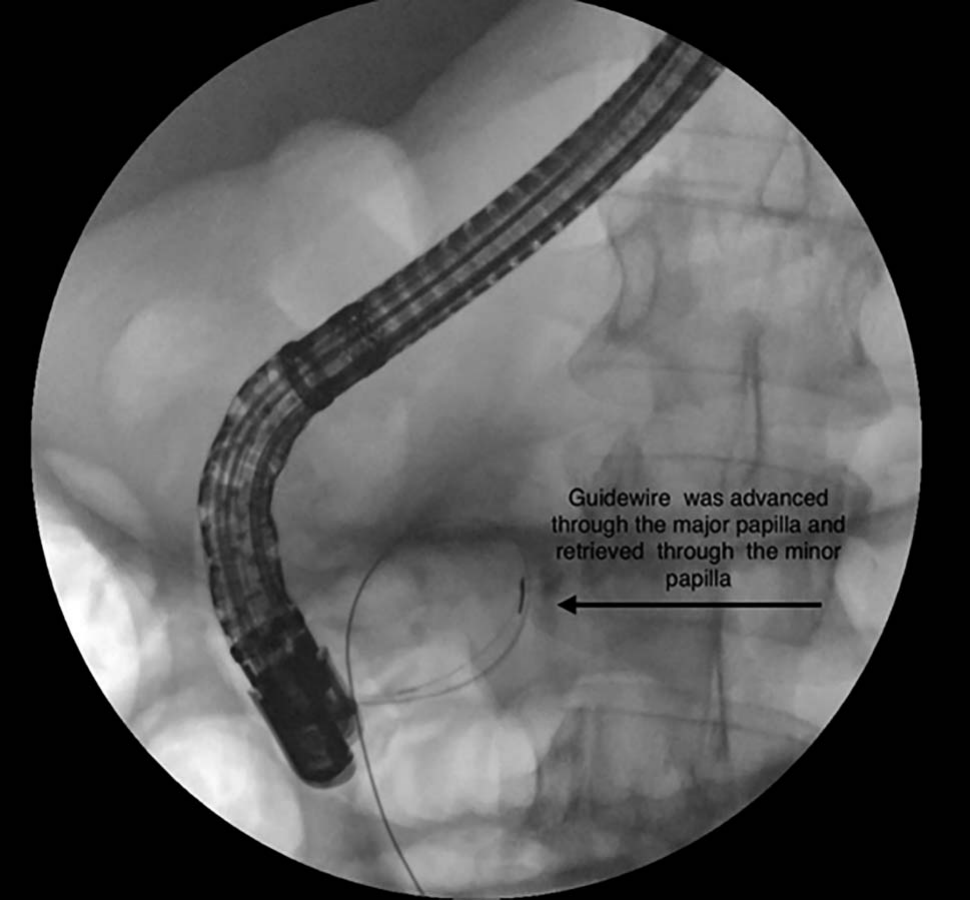
Duodenoscopic-assisted completion of the transpapillary rendezvous technique.

**Figure 3.**
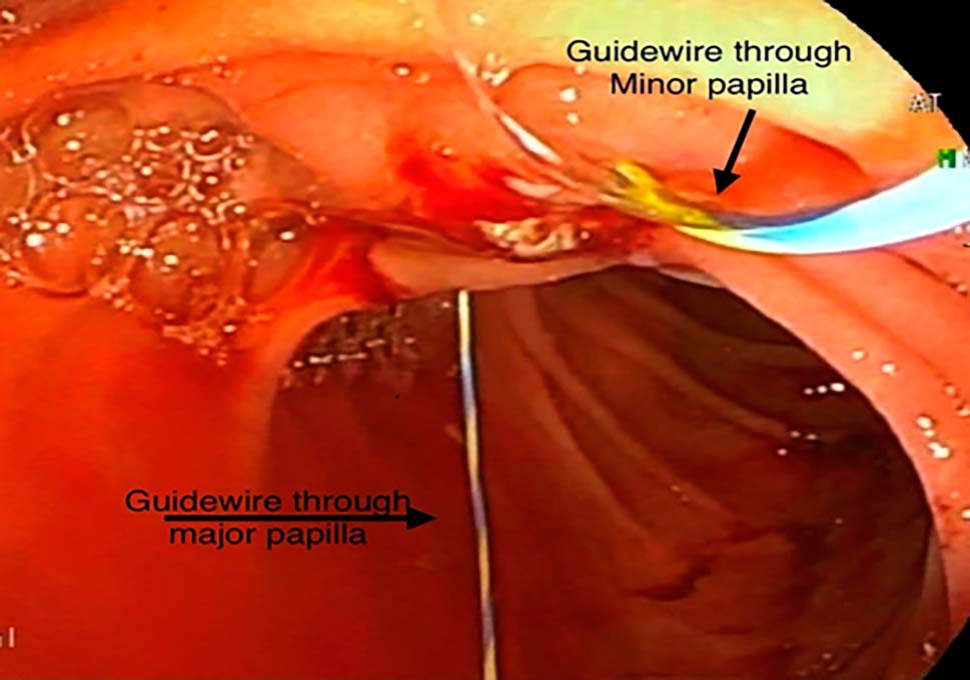
Guidewire retrieval through the minor papilla with access to the dorsal pancreatic duct.

A minor papilla sphincterotomy was subsequently performed, followed by spontaneous passage of the impacted pancreatic duct stone through the sphincterotomy without the need for balloon extraction (Figure 4). A 5 Fr × 7 cm single-pigtail plastic stent was then successfully placed within the dorsal pancreatic duct to facilitate pancreatic drainage (Figure 5). No postsphincterotomy bleeding or immediate procedure-related complications were observed. Following the intervention, the patient reported complete resolution of her abdominal pain. She was discharged home within 24 hours on pancreatic enzyme supplementation, with outpatient follow-up arranged.

**Figure 4.**
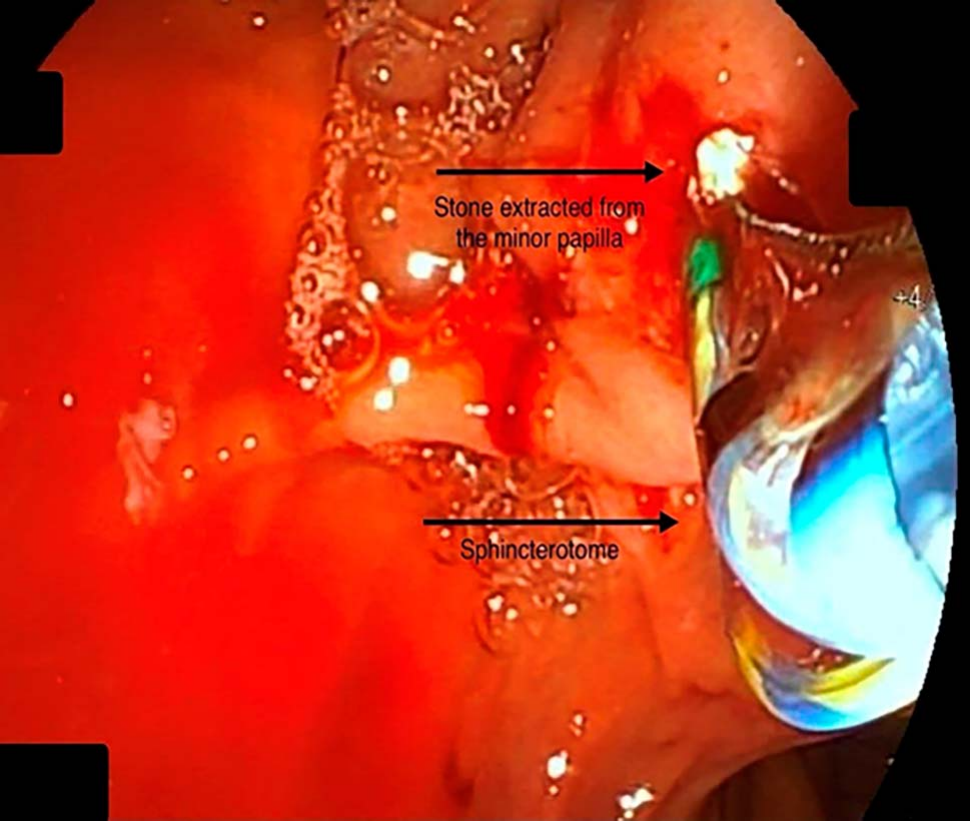
Minor papilla sphincterotomy and extraction of the impacted pancreatic duct stone.

**Figure 5.**
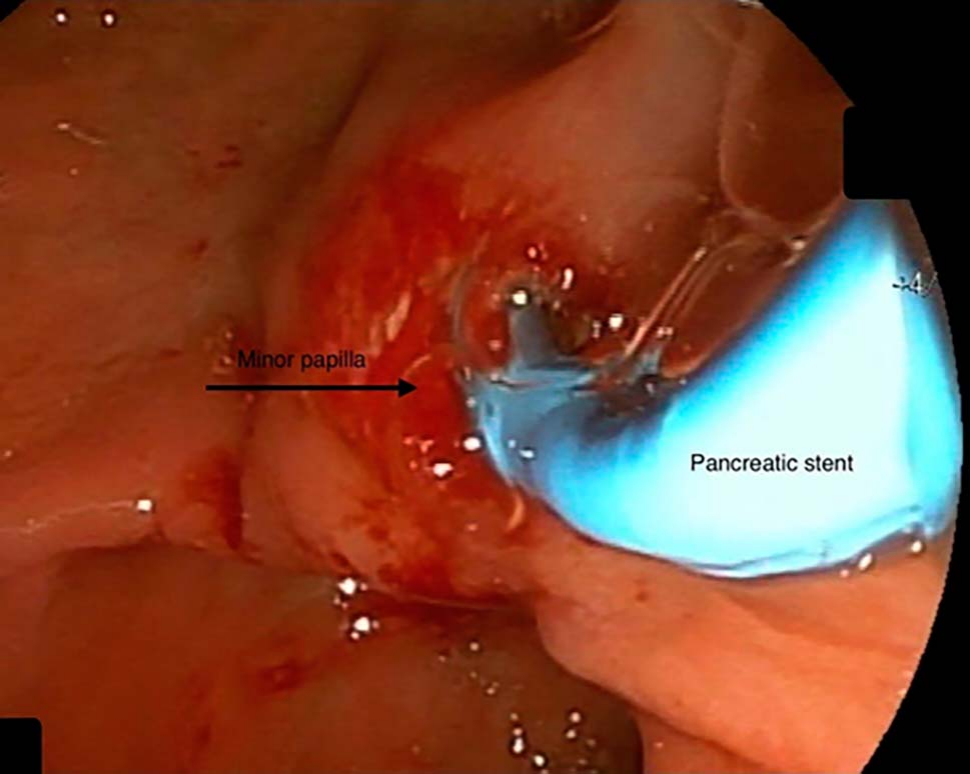
Successful placement of a single-pigtail plastic stent in the dorsal pancreatic duct.

## DISCUSSION

PD is the most common congenital anomaly of the pancreatic ductal system, resulting from incomplete fusion of the dorsal and ventral pancreatic ducts during embryologic development, with an estimated prevalence of 5%–10% in the general population.^4,5^ In incomplete PD, a small communicating branch persists between the dorsal and ventral ductal systems, although the majority of pancreatic drainage occurs through the minor papilla.^3,4^ While most individuals with PD remain asymptomatic, impaired drainage through the minor papilla and relative outflow obstruction have been proposed as potential mechanisms predisposing susceptible individuals to recurrent acute or chronic pancreatitis.^3,5^

In this case, initial computed tomography and MRI/MRCP demonstrated established chronic pancreatitis with obstructing pancreatic ductal calculi but did not identify a congenital ductal anomaly. EUS provided additional anatomical information by demonstrating apparent drainage of the pancreatic ductal system through the minor papilla, raising suspicion for PD. This illustrates the complementary role of EUS in evaluating pancreatic ductal anatomy when cross-sectional imaging is inconclusive, particularly in patients with recurrent pancreatitis and persistent symptoms.

Cannulation of the minor papilla remains one of the most technically challenging aspects of ERCP in patients with PD.^3,4^ The challenge is further increased when the minor papillary orifice is diminutive or stenotic, as in our patient. Conventional cannulation and guidewire advancement into the dorsal pancreatic duct were unsuccessful. A transpapillary rendezvous technique was therefore employed, whereby a guidewire was advanced through the major papilla and across a small communicating branch between the ventral and dorsal pancreatic ducts toward the minor papilla. Retrieval of the guidewire through the minor papilla subsequently established access to the dorsal pancreatic duct, allowing minor papilla sphincterotomy, spontaneous passage of the obstructing pancreatic duct stone, and pancreatic stent placement. This approach provided definitive endoscopic therapy while avoiding surgical intervention and demonstrates how detailed knowledge of pancreatic ductal anatomy can facilitate successful management of technically challenging ERCP procedures.^6–8^

Endoscopic therapy is generally considered the preferred initial treatment of symptomatic PD.^4,5,9^ Minor papilla sphincterotomy, with or without temporary pancreatic duct stenting, has demonstrated potential benefit in selected patients with recurrent acute pancreatitis, whereas outcomes tend to be less favorable in patients with established chronic pancreatitis, likely because of irreversible parenchymal fibrosis and structural pancreatic damage.^4,5,9^ In our patient, however, the clinical context differed from isolated PD. She had established chronic calcific pancreatitis with an impacted pancreatic duct stone and evidence of ductal obstruction, providing a clear therapeutic target for ductal decompression. Restoration of dorsal ductal drainage and spontaneous passage of the obstructing stone were followed by complete resolution of her longstanding abdominal pain.

When conventional minor papilla cannulation is unsuccessful, several advanced rescue strategies have been described. EUS-guided rendezvous techniques can provide alternative access to the pancreatic duct but require advanced expertise and may increase procedural complexity.^6,7^ In contrast, transpapillary rendezvous approaches that exploit the communicating branch between the ventral and dorsal ducts in incomplete PD have been rarely reported.^8^ Tsuji et al^8^ described a rendezvous precut technique for incomplete PD with successful technical outcomes. Our case adds to this limited body of evidence by demonstrating that a transpapillary rendezvous approach can overcome failed minor papilla cannulation and facilitate access to the dorsal pancreatic duct without requiring an EUS-guided intervention.

An important feature of this case is that incomplete PD was recognized during ERCP despite the absence of a clearly identified ductal anomaly on the initial MRI/MRCP examination. Identification of the communicating branch between the ventral and dorsal pancreatic ducts not only established the diagnosis but also provided the route through which the rendezvous technique could be performed. This underscores the importance of careful fluoroscopic assessment of pancreatic ductal anatomy during ERCP, particularly when the expected anatomy is not encountered and conventional cannulation is unsuccessful.

To the best of our knowledge, and following a comprehensive review of the available literature, this represents the first reported case from Lebanon describing successful endoscopic management of incomplete PD using a transpapillary rendezvous-assisted ERCP technique after failed conventional minor papilla cannulation. Although similar rescue approaches have been reported internationally, the published experience remains limited and consists largely of isolated case reports.^6–8^ Our case therefore contributes additional clinical experience regarding the feasibility of this technically demanding approach.

The principal strength of this case is the successful application of an uncommon endoscopic rescue technique in a challenging anatomical setting. Despite a markedly diminutive and edematous minor papilla, the procedure achieved access to the dorsal pancreatic duct, allowing successful sphincterotomy, passage of the obstructing stone, and pancreatic stent placement. More broadly, the case emphasizes the importance of recognizing pancreatic ductal anatomical variants and adapting the endoscopic strategy to the underlying anatomy and mechanism of obstruction.

Several limitations should be acknowledged. First, as a single-case report, the findings cannot be generalized to all patients with PD or chronic pancreatitis. Second, long-term clinical outcomes following the intervention remain unknown. Finally, because most individuals with PD remain asymptomatic,^3–5^ a causal relationship between incomplete PD and this patient's chronic pancreatitis cannot be established with certainty. Other etiologic factors, including genetic predisposition, may have contributed to her disease. Nevertheless, impaired drainage of the dorsal pancreatic duct, compounded by an obstructing pancreatic duct stone, likely played an important role in her clinical presentation.

The recent SHARP randomized clinical trial further emphasizes the need for caution when attributing therapeutic benefit to endoscopic intervention performed solely on the basis of PD. The trial found that minor papillotomy did not significantly reduce the risk of recurrent acute pancreatitis in patients with unexplained recurrent acute pancreatitis and PD, challenging the assumption that correction of minor papillary outflow obstruction alone necessarily translates into clinical benefit.^10^ Importantly, patients with concomitant chronic calcific pancreatitis were excluded from the SHARP trial. Therefore, its findings should not be directly extrapolated to patients with established chronic pancreatitis and a demonstrable obstructive mechanism, in whom endoscopic therapy may be directed toward ductal decompression and removal of an obstructing stone or stricture. In this context, the therapeutic benefit observed in our patient should be interpreted primarily in relation to relief of mechanical pancreatic ductal obstruction rather than attributed solely to the presence of PD.^10^

Collectively, this case highlights the importance of considering PD in patients with recurrent pancreatitis, particularly when pancreatic ductal anatomy is atypical or conventional cannulation is unexpectedly difficult. In patients with incomplete PD and failed minor papilla cannulation because of a diminutive or stenotic orifice, a transpapillary rendezvous technique may represent a feasible, minimally invasive rescue strategy for obtaining dorsal pancreatic duct access and facilitating definitive endoscopic therapy.^6–8^ Further experience and larger studies are needed to better define the technical success, clinical efficacy, and long-term outcomes of this uncommon approach.

This case highlights incomplete PD as a clinically relevant pancreatic ductal anomaly that should be considered in patients with recurrent or otherwise unexplained pancreatitis, particularly when atypical ductal anatomy or pancreatic ductal obstruction is encountered. It underscores the importance of careful assessment of pancreatic ductal anatomy and the complementary role of cross-sectional imaging, EUS, and ERCP in establishing the diagnosis and guiding management.

In selected symptomatic patients, endoscopic therapy may restore pancreatic ductal drainage and provide meaningful symptomatic relief, particularly when a demonstrable obstructive mechanism is present. Although EUS-guided rendezvous techniques have become increasingly used in expert centers, a transpapillary rendezvous approach may provide a valuable alternative when conventional minor papilla cannulation is unsuccessful. In this case, this minimally invasive technique enabled successful access to the dorsal pancreatic duct, facilitating sphincterotomy, spontaneous passage of the obstructing stone, and pancreatic stent placement while avoiding surgical intervention. This case therefore emphasizes the importance of tailoring the endoscopic approach to the individual patient's ductal anatomy and underlying mechanism of obstruction, while recognizing that the clinical significance of PD itself remains variable.

## DISCLOSURES

Author contributions: R. Radwan, D. Siblini, and R. Siblini contributed substantially and comparably to the conception and design of the work, literature review, data collection, clinical data interpretation, and drafting and revision of the manuscript. A. Rachid and Z. Houmani supervised the clinical and scientific aspects of the work, provided critical intellectual input, and contributed to the interpretation of the findings and critical revision of the manuscript. A. Rachid additionally coordinated the manuscript preparation and served as the corresponding author. All authors reviewed and approved the final version of the manuscript and agree to be accountable for all aspects of the work, ensuring that questions related to the accuracy or integrity of any part of the work are appropriately investigated and resolved.

Financial disclosure: None to report.

Informed consent was obtained for this case report.

## ABBREVIATIONS:

ERCP: endoscopic retrograde cholangiopancreatography
EUS: endoscopic ultrasound
MRCP: Magnetic resonance cholangiopancreatography
MRI: magnetic resonance imaging
PD: Pancreas divisum
